# Supplementary figures and images for: Molecular mechanisms of fentanyl mediated β-arrestin biased signaling
Source: PLoS Comput Biol. 2020 Apr 10;16(4):e1007394. doi: 10.1371/journal.pcbi.1007394 (PMC7176292; doi:10.1371/journal.pcbi.1007394)

**S1 Fig.****a**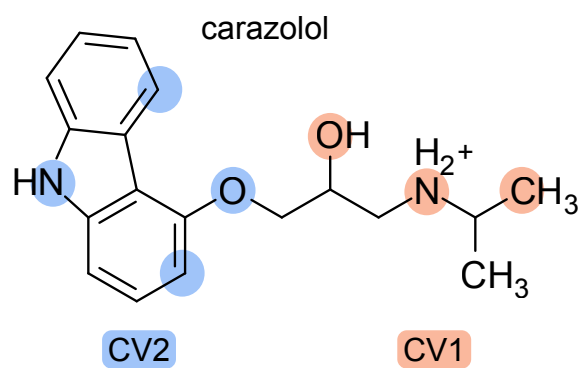**b**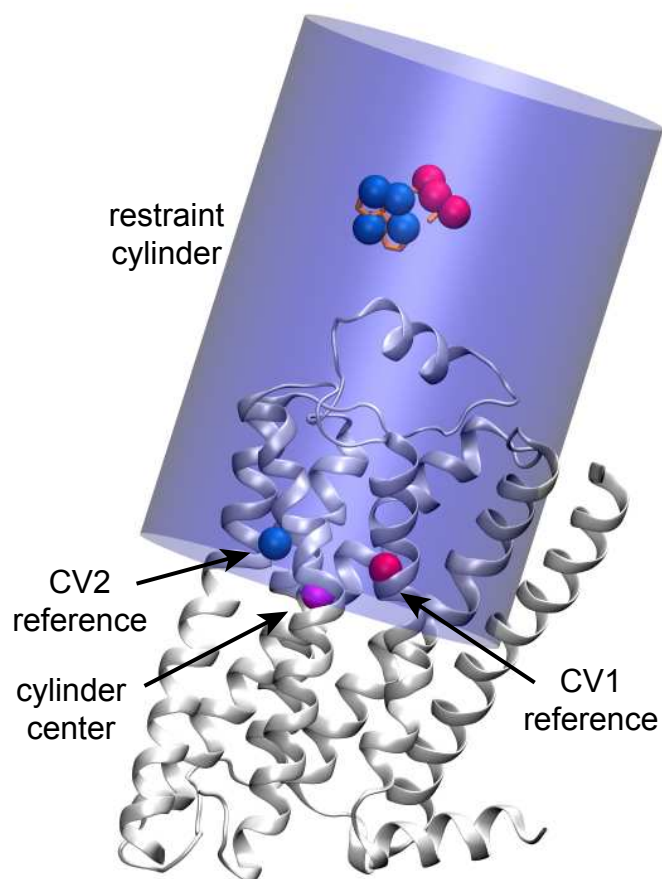**c**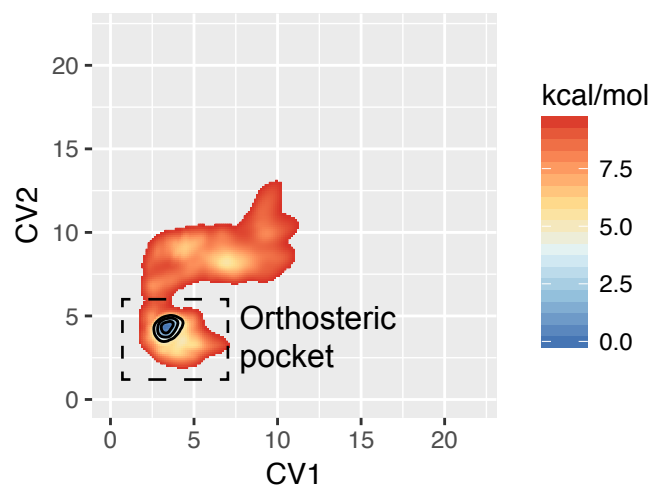**d**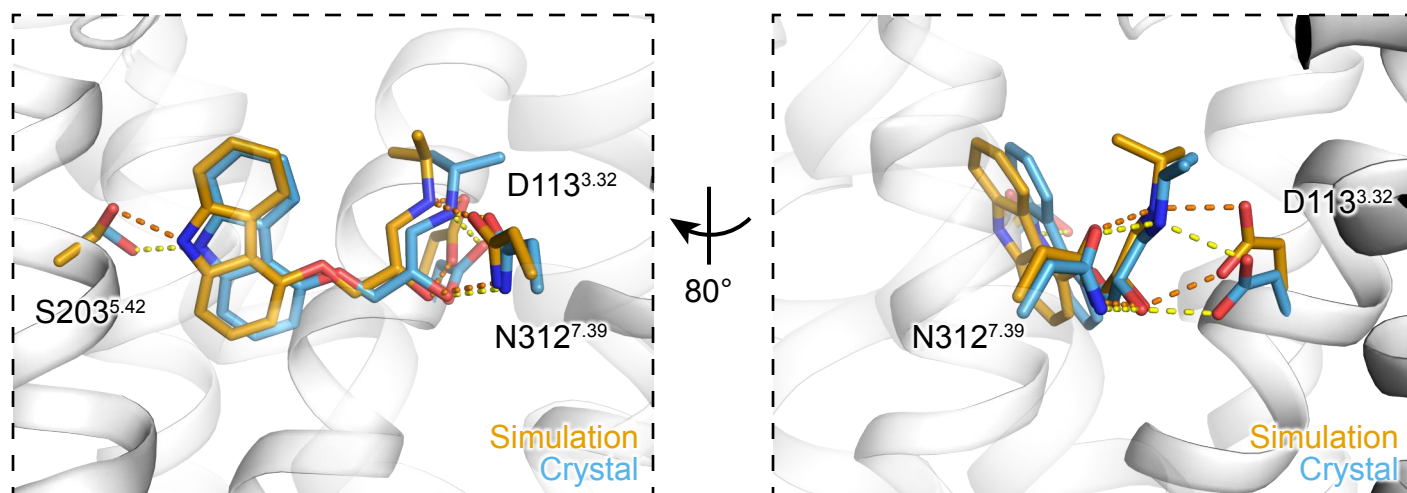

Supplement: S1 Fig — a-b, graphical representation of collective variables and cylindrical restraints used in mABP simulations. c, Averaged free energy landscape for carazolol binding to the β2AR. Contours are drawn at 1, 3, and 5 kcal/mol. d, Structural overlap of carazolol’s predicted low-energy conformation (white cartoon; orange sticks) and crystal structure (PDB Code: 2RH1; light blue sticks). (PDF) [file pcbi.1007394.s001.pdf]

**S2 Fig.****a**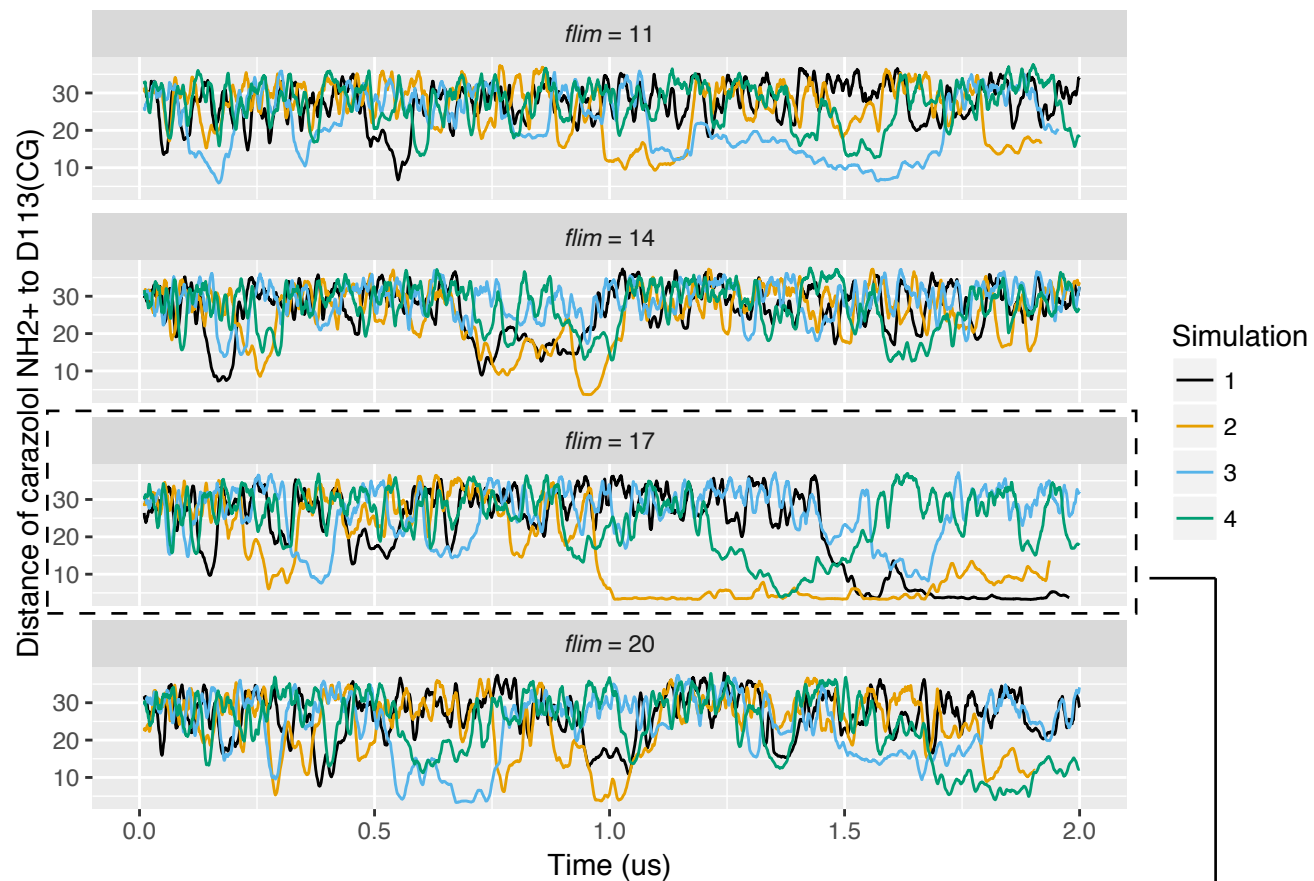**b**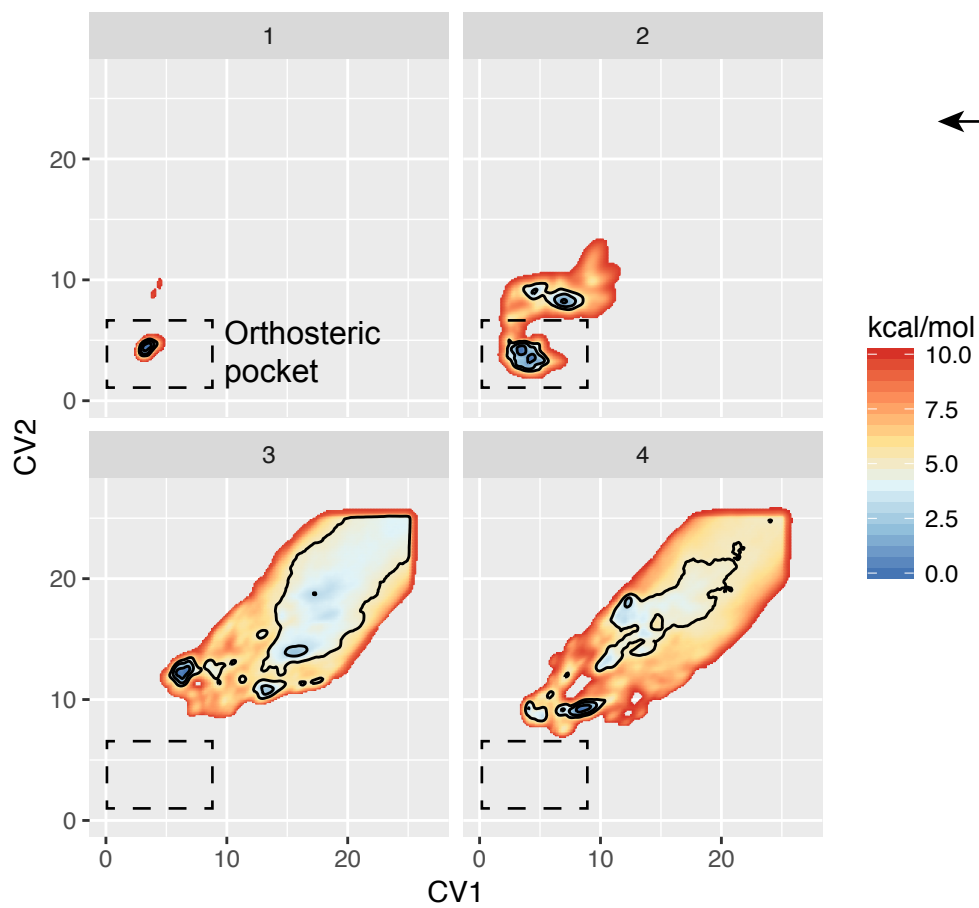

Supplement: S2 Fig — a, Distance between carazolol’s protonated amine to D1133.32 for various bias potential fill-limits. b, Free energy landscapes of mABP simulations where the bias potential was capped at 17 kcal/mol. Contours are drawn at 1, 3, and 5 kcal/mol. (PDF) [file pcbi.1007394.s002.pdf]

S3 Fig.

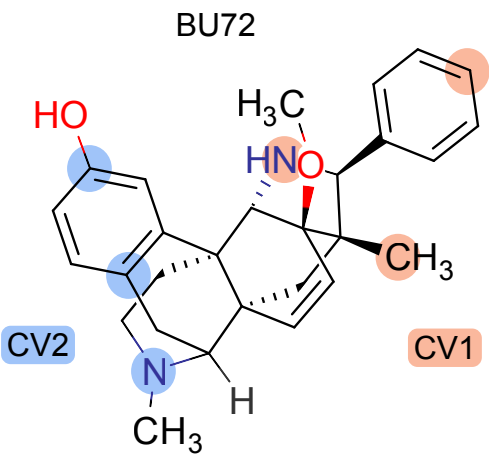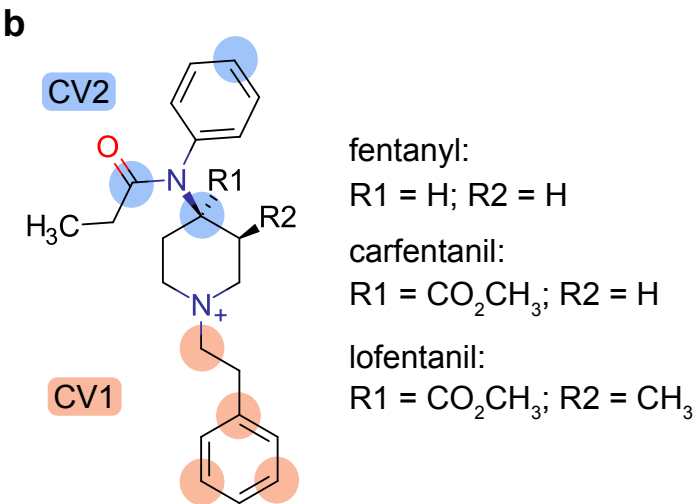

Supplement: S3 Fig — a-b, Ligand atom selections for CV1 and CV2 for BU72 and fentanyl. (PDF) [file pcbi.1007394.s003.pdf]

S4 Fig.

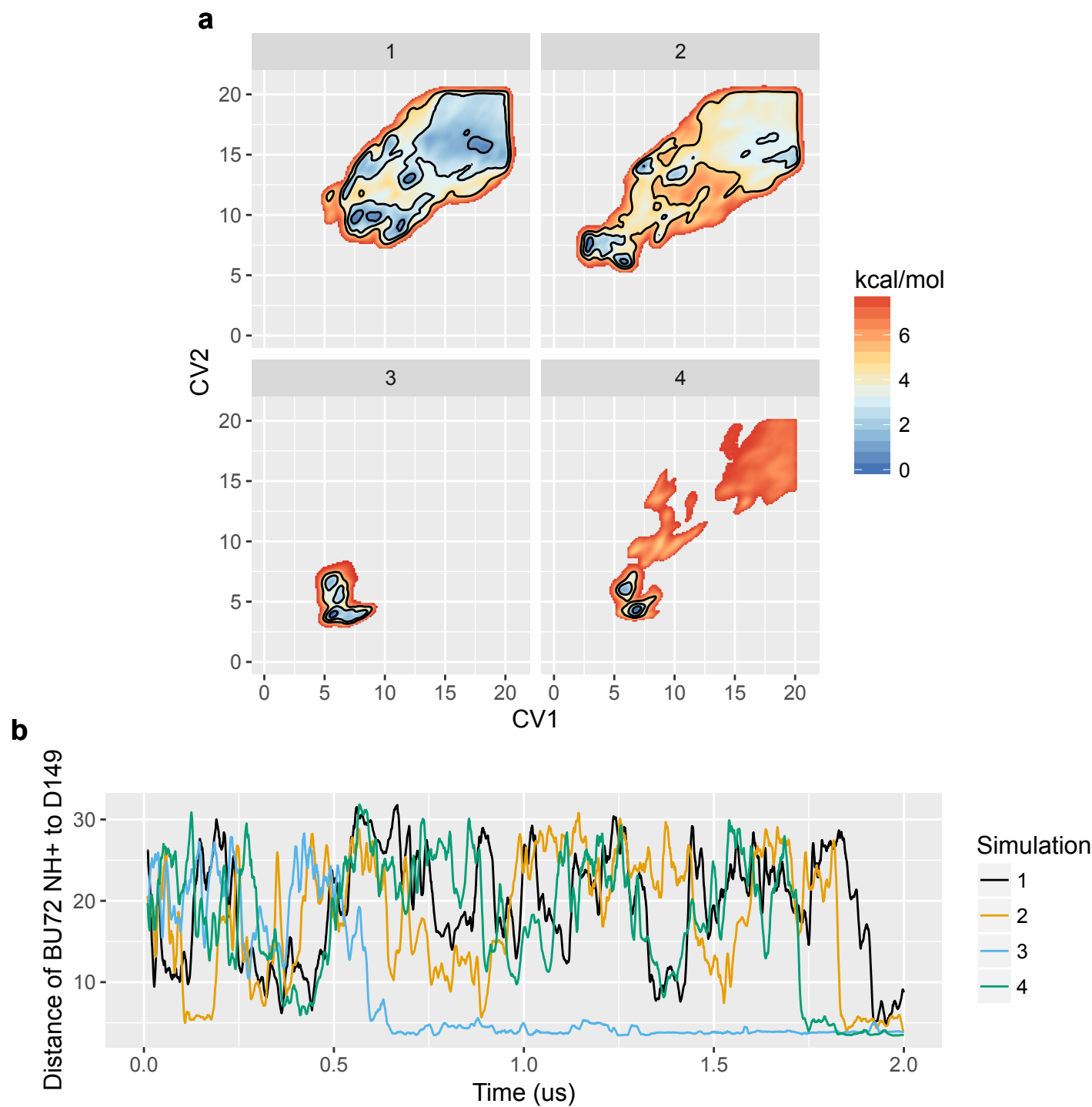

Supplement: S4 Fig — a, Free energy landscapes of simulation replicates. Contours are drawn at 1, 3, and 5 kcal/mol. b, Distance between BU72’s protonated amine to D1493.32. (PDF) [file pcbi.1007394.s004.pdf]

S5 Fig.

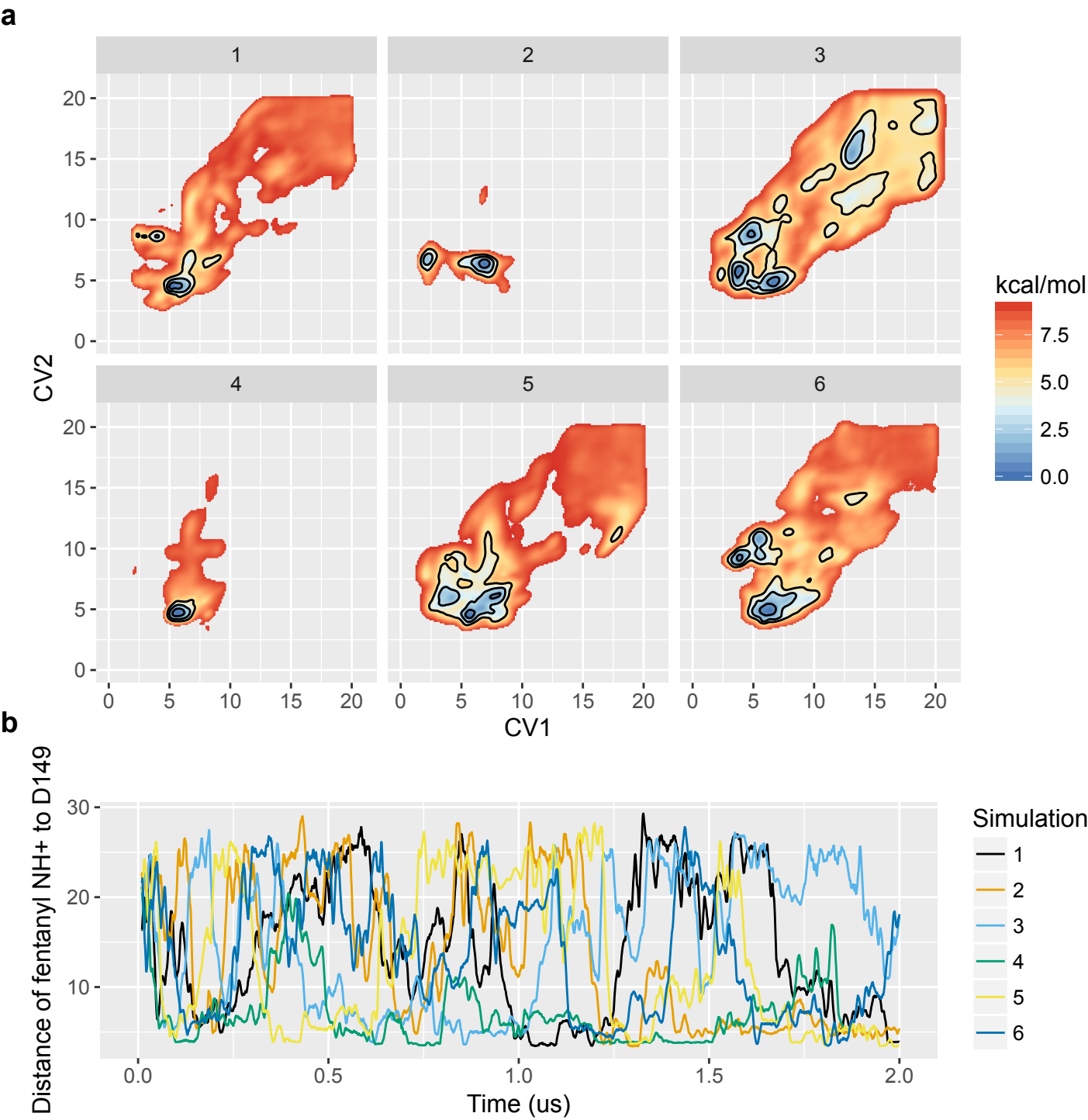

Supplement: S5 Fig — a, Free energy landscapes of simulation replicates. Contours are drawn at 1, 3, and 5 kcal/mol. b, Distance between fentanyl’s protonated amine to D1493.32. (PDF) [file pcbi.1007394.s005.pdf]

S6 Fig.

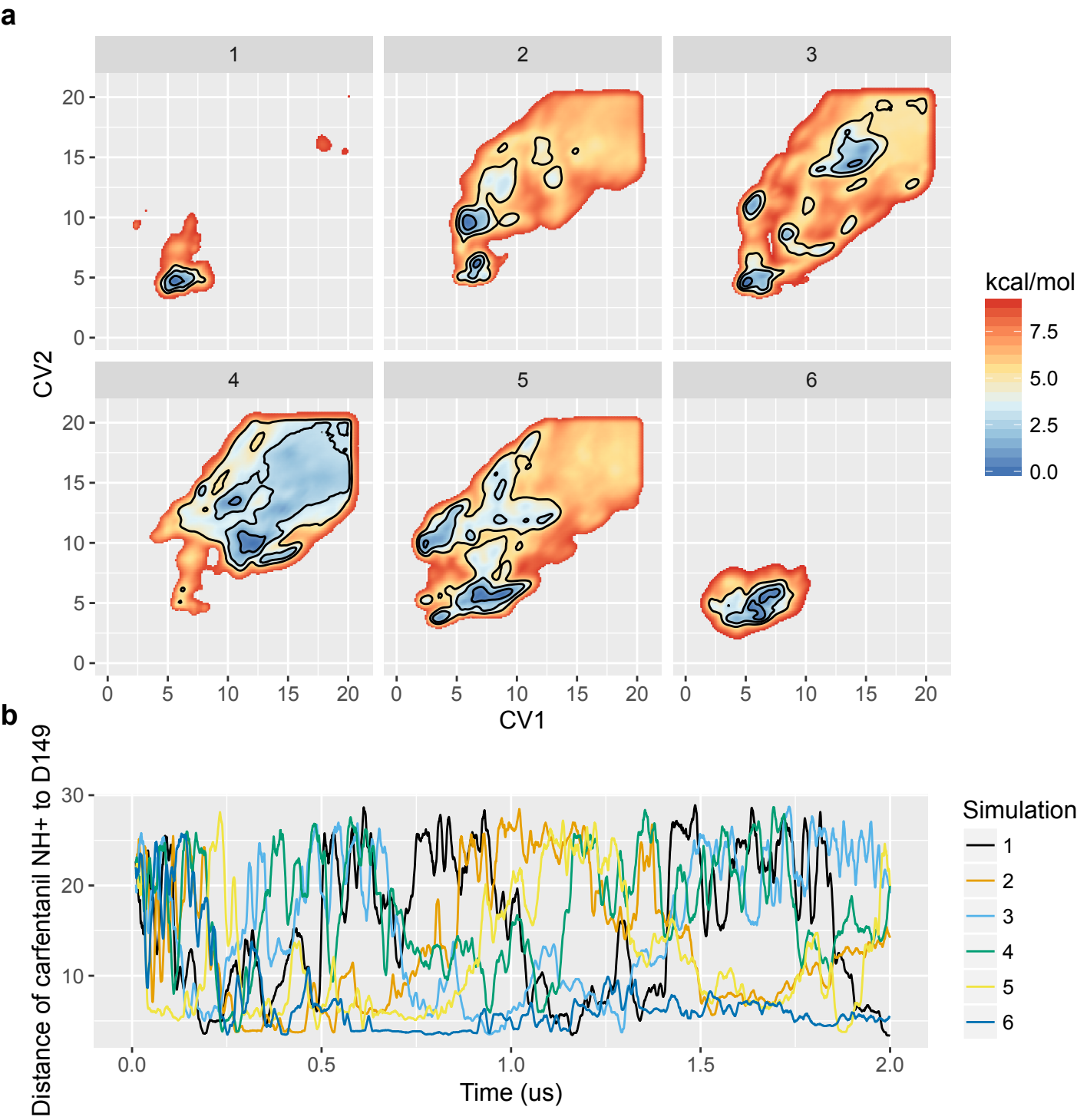

Supplement: S6 Fig — a, Free energy landscapes of simulation replicates. Contours are drawn at 1, 3, and 5 kcal/mol. b, Distance between carfentanil’s protonated amine to D1493.32. (PDF) [file pcbi.1007394.s006.pdf]

S7 Fig.

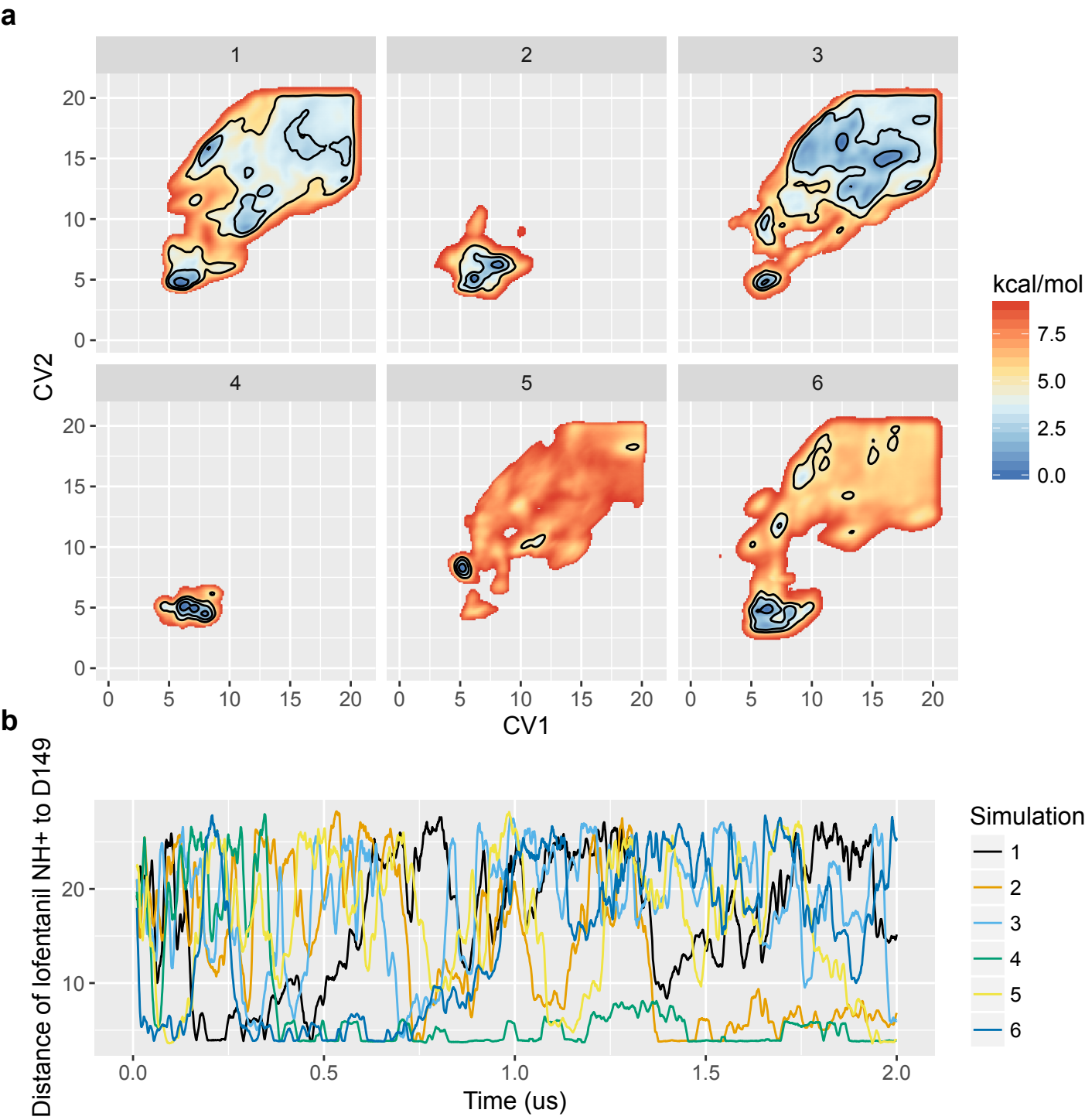

Supplement: S7 Fig — a, Free energy landscapes of simulation replicates. Contours are drawn at 1, 3, and 5 kcal/mol. b, Distance between lofentanil’s protonated amine to D1493.32. (PDF) [file pcbi.1007394.s007.pdf]

S8 Fig.

a

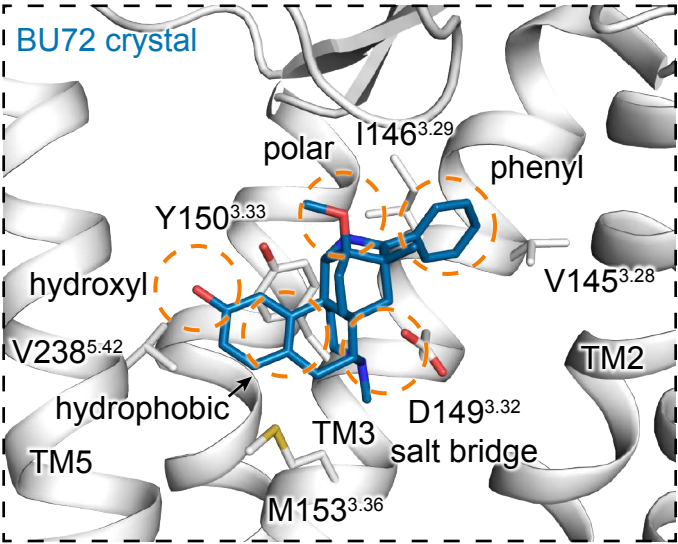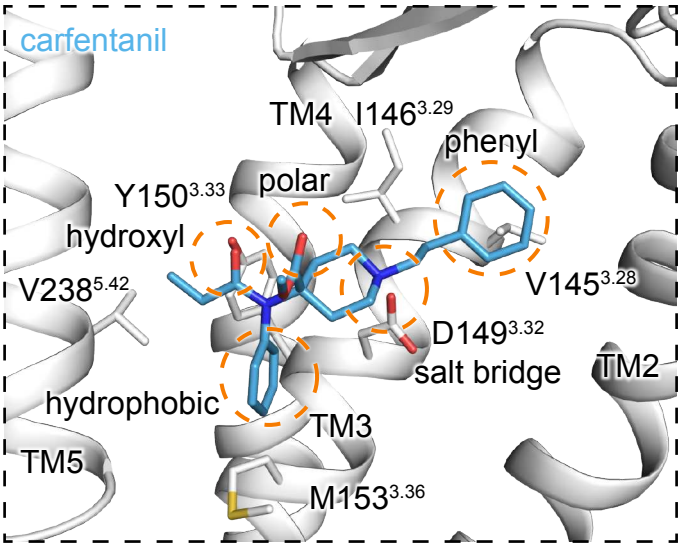

Supplement: S8 Fig — a, Side-by-side structural and pharmacophore comparison between BU72 (PDB code: 5C1M) and carfentanil bound μOR. (PDF) [file pcbi.1007394.s008.pdf]

S9 Fig.

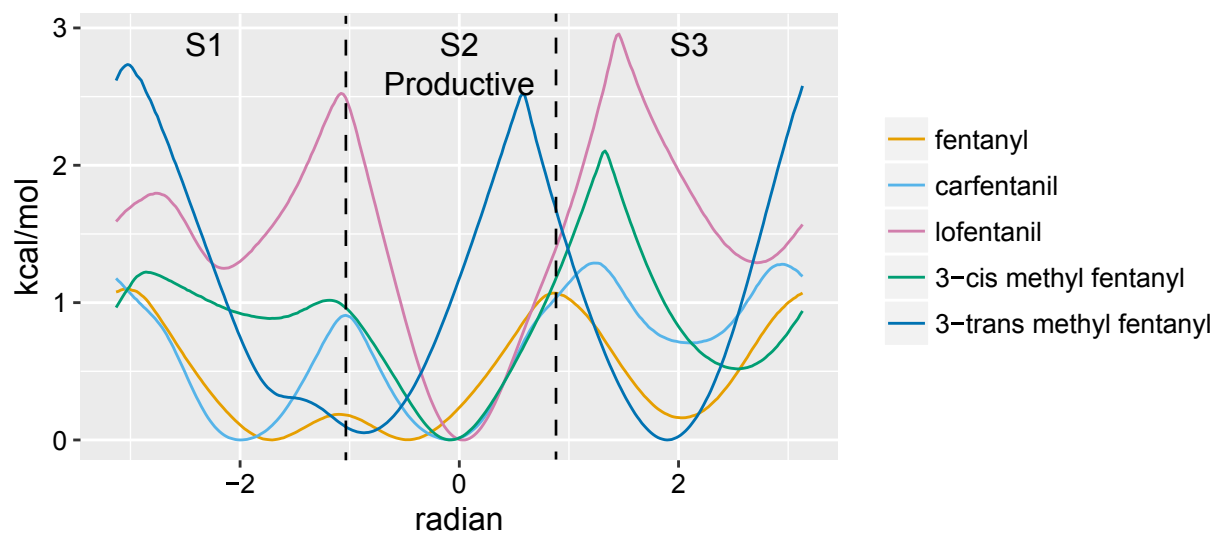

Supplement: S9 Fig — a, S2 dihedral occupancy for fentanyl, carfentanil, and lofentanil, 3-cis methyl fentanyl, and 3-trans methyl fentanyl was calculated to be 38.5%, 43.4%, 75.9%, 56.3%, and 34.7% respectfully. (PDF) [file pcbi.1007394.s009.pdf]

S10 Fig.

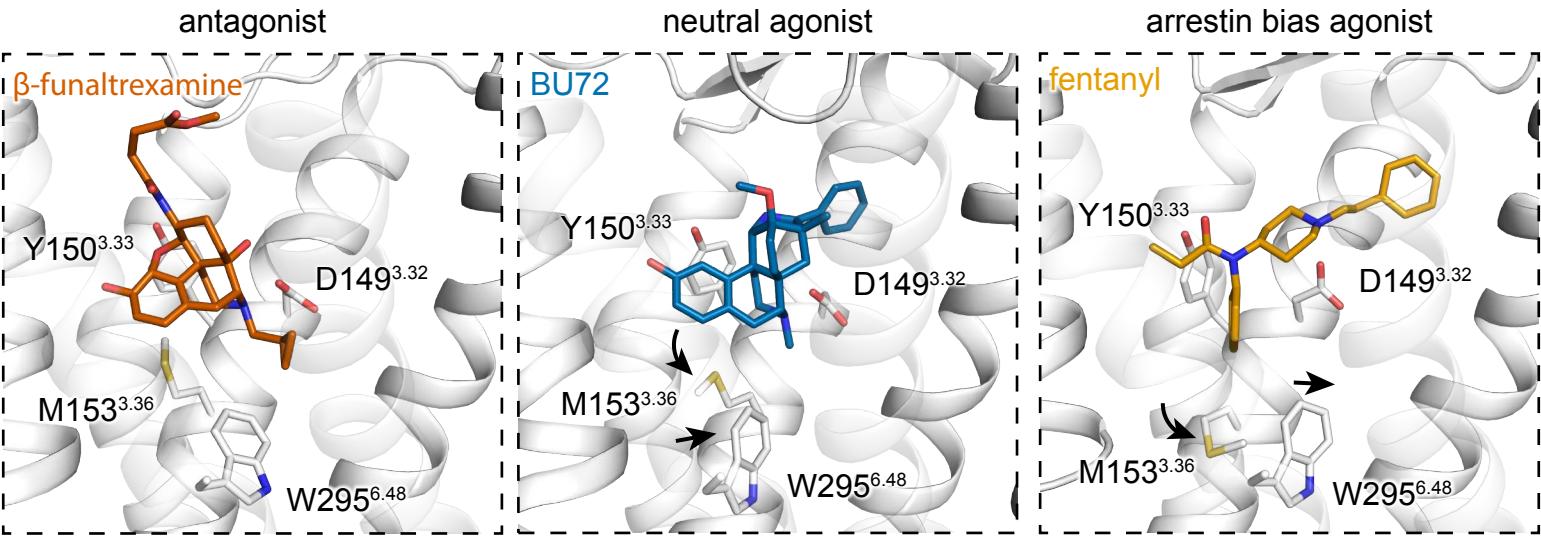

Supplement: S10 Fig — a, Side-by-side comparison of antagonist (PDB code: 4DKL), agonist (PDB code: 5C1M), and predicted fentanyl bound pose. M1533.36 and W2956.48 side-chain rearrangements are annotated. (PDF) [file pcbi.1007394.s010.pdf]

**S11 Fig.**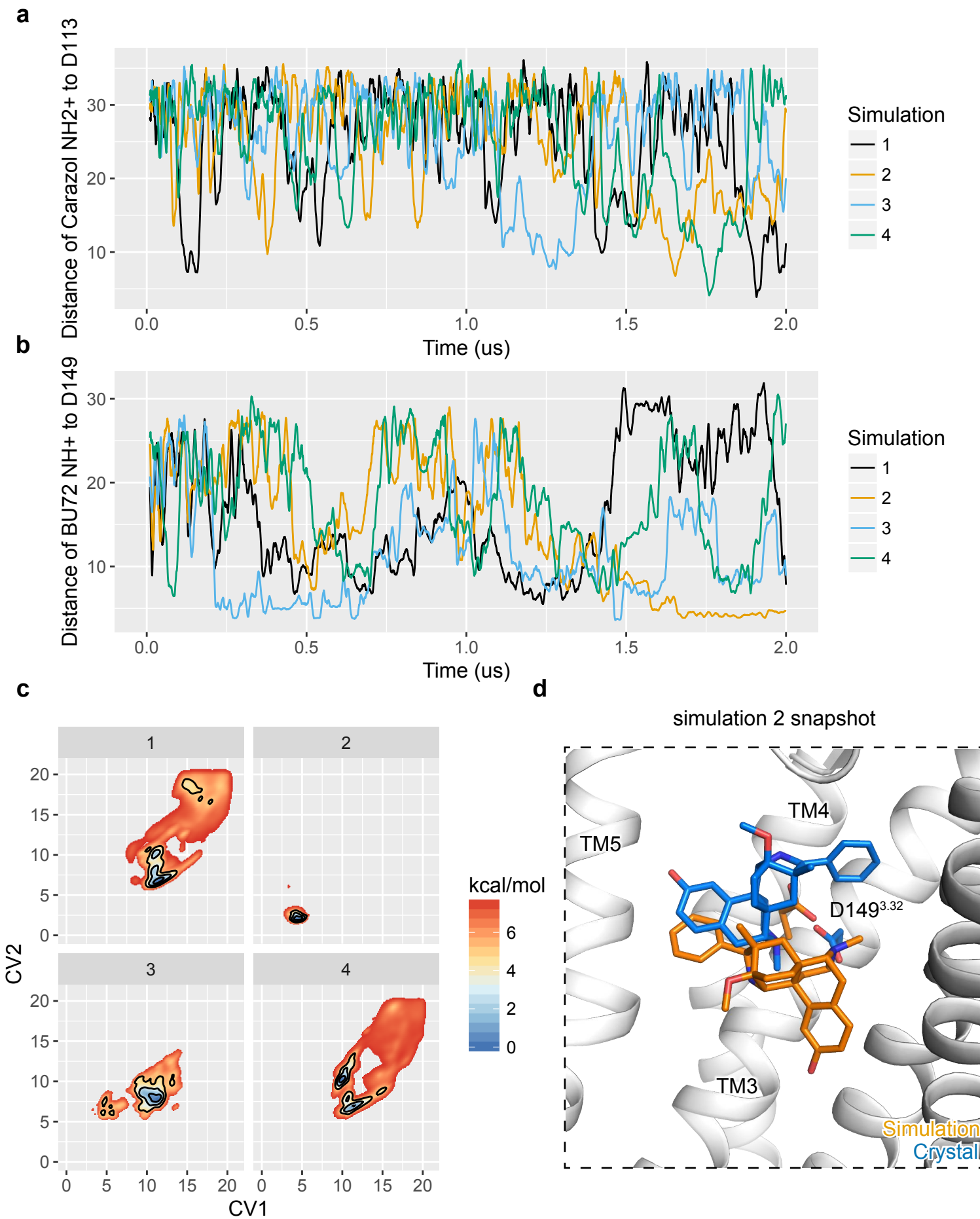

Supplement: S11 Fig — a, Distance between carazolol’s protonated amine to D1133.32. b, Distance between BU72’s protonated amine to D1493.32. c, Free energy landscapes of for BU72 simulations with switched CV reference points. Contours are drawn at 1, 3, and 5 kcal/mol. d, Structural comparison of the most populated conformation of simulation 2 compared to the crystal structure of BU72. (PDF) [file pcbi.1007394.s011.pdf]

**S12 Fig.**

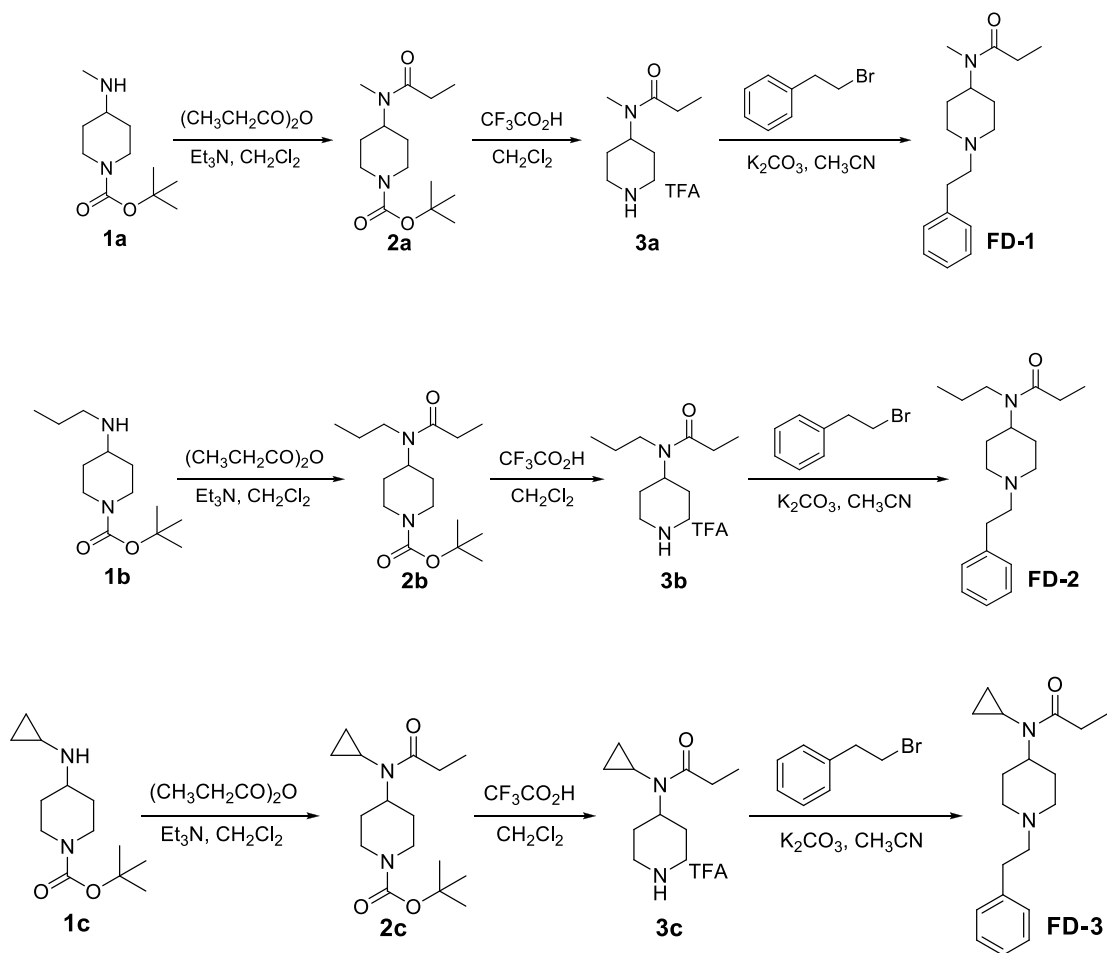

Supplement: S12 Fig — (PDF) [file pcbi.1007394.s012.pdf]
