# Supplementary material for: Molecular mechanisms of fentanyl mediated β-arrestin biased signaling
Source: PLoS Comput Biol. 2020 Apr 10;16(4):e1007394. doi: 10.1371/journal.pcbi.1007394 (PMC7176292; doi:10.1371/journal.pcbi.1007394)
Supplement: S1 Table — * Indicates simulations where CVs are switched. (DOCX) [file pcbi.1007394.s013.docx]

| Receptor | Ligand | Atoms | Dimensions (Å) | flim (kcal/mol) | replicates | Length (total; μs) |
| --- | --- | --- | --- | --- | --- | --- |
| β2AR (2rh1) | carazolol | 57496 | 87 x 87 x 89 | 11 | 1-4 | 2 (8) |
| β2AR (2rh1) | carazolol | 57496 | 87 x 87 x 89 | 14 | 1-4 | 2 (8) |
| β2AR (2rh1) | carazolol | 57496 | 87 x 87 x 89 | 17 | 1-4, 5-8* | 2 (16) |
| β2AR (2rh1) | carazolol | 57496 | 87 x 87 x 89 | 20 | 1-4 | 2 (8) |
| μOR (4dkl) | BU72 | 52398 | 79 x 79 x95 | 15 | 1-4, 5-8* | 2 (16) |
| μOR (4dkl) | fentanyl | 52405 | 79 x 79 x95 | 15 | 1-6 | 2 (12) |
| μOR (4dkl) | carfentanil | 52411 | 79 x 79 x95 | 15 | 1-6 | 2 (12) |
| μOR (4dkl) | lofentanil | 52414 | 79 x 79 x95 | 15 | 1-6 | 2 (12) |
| N/A | fentanyl | 2320 | 31 x 31 x 31 | N/A | 1 | 0.5 |
| N/A | carfentanil | 2374 | 31 x 31 x 31 | N/A | 1 | 0.5 |
| N/A | lofentanil | 2377 | 31 x 31 x 31 | N/A | 1 | 0.5 |
| N/A | 3-cmf | 2317 | 31 x 31 x 31 | N/A | 1 | 0.5 |
| N/A | 3-tmf | 3415 | 33 x 33 x 33 | N/A | 1 | 0.5 |
