# Supplementary material for: Molecular mechanisms of fentanyl mediated β-arrestin biased signaling
Source: PLoS Comput Biol. 2020 Apr 10;16(4):e1007394. doi: 10.1371/journal.pcbi.1007394 (PMC7176292; doi:10.1371/journal.pcbi.1007394)
Supplement: S2 Table — Binding events, including both ligand binding and unbinding from the receptor, were determined by an interatom distance between the carboxylic acid carbon on Asp3.32 and the protonated amine found on each ligand. To be considered bound the inter-atom distance must be less than 4 Å, and to be considered unbound the inter-atom distance must be greater than 10 Å. For carazolol, we computed efficiency based on simulations 1–4 (flim 17 kcal/mol). For BU72, we computed efficiency based on simulations 1–4. For Dror et al., binding events included poses 4, 4’, 4”, and 5 as defined in [25]. (DOCX) [file pcbi.1007394.s014.docx]

| Simulation  Type | System | Ligand | Aggregate simulation time (μs) | Binding events | Binding events per μs | Reference |
| --- | --- | --- | --- | --- | --- | --- |
| Biased | β2AR | carazolol | 8 | 10 | 1.2636 | This work |
| Biased | μOR | BU72 | 8 | 13 | 1.6250 | This work |
| Biased | μOR | fentanyl | 12 | 66 | 5.5676 | This work |
| Biased | μOR | carfentanil | 12 | 59 | 3.9058 | This work |
| Biased | μOR | lofentanil | 12 | 40 | 3.3333 | This work |
| Classical | β2AR | dihydroalprenolol (condition A) | 71.3 | 7 | 0.0982 | Dror et al.[1] |
| Classical | β2AR | dihydroalprenolol (condition B) | 40.5 | 3 | 0.0741 | Dror et al. [1] |
| Classical | β2AR | alprenolol | 14 | 2 | 0.1429 | Dror et al. [1] |
| Classical | β2AR | propranolol | 35.7 | 3 | 0.0840 | Dror et al. [1] |
| Classical | β2AR | isoproterenol | 15 | 1 | 0.0667 | Dror et al. [1] |
| Classical | β2AR | dihydroalprenolol | 55.5 | 5 | 0.0901 | Dror et al. [1] |

Reference:

1. Dror RO, Pan AC, Arlow DH, Borhani DW, Maragakis P, Shan Y, et al. Pathway and mechanism of drug binding to G-protein-coupled receptors. PNAS. 2011;108(32):13118-23. doi: 10.1073/pnas.1104614108.
