# Supplementary material for: Molecular mechanisms of fentanyl mediated β-arrestin biased signaling
Source: PLoS Comput Biol. 2020 Apr 10;16(4):e1007394. doi: 10.1371/journal.pcbi.1007394 (PMC7176292; doi:10.1371/journal.pcbi.1007394)
Supplement: Attachment — Submitted filename: review2.pdf [file pcbi.1007394.s019.pdf]

As a preliminary note, I must compare the review sent by Reviewer 1 and the abstract of the manuscript:

Reviewer #1	Abstract
Reviewer #1: *This is very important and well written report describing in the first time proper structure-based drug-design of GPCR biased agonists. Such ligands hold high therapeutic potential as being more efficient and clean from side-effects. It is very promising area however very challenging. The key challenge is to isolate the key features in GPCR binding site responsible for G-protein or β-arrestin signalling/activation pathway (via binding of the agonist).* *The authors used adaptively biased molecular dynamics simulations to predict which chemical features dictate G protein or β-arrestin signalling.* ***The resulting fentanyl-bound pose provides rational insight into a wealth of historical structure-activity-relationship on its chemical scaffold. Authors found that fentanyl and the synthetic opioid peptide DAMGO require M153 to induce β-arrestin coupling, while M153 was dispensable for G protein coupling. We propose and validate a mechanism where the n-aniline ring of fentanyl mediates μOR β-arrestin through a novel M153 "microswitch" by synthesizing fentanyl-based derivatives that exhibit complete, clinically desirable, G protein biased coupling.*** *Such approach is pioneering and it is great to see that it is working. It is very timely report that would be interesting to wide audience of drug-discovery researches and encourage them to apply such approach in their research.* *My recommendation will be to publish it as it is.*	***Abstract*** *The development of novel analgesics with improved safety profiles to combat the opioid epidemic represents a central question to G protein coupled receptor structural biology and pharmacology: What chemical features dictate G protein or β-arrestin signaling? Here we use adaptively biased molecular dynamics simulations to determine how fentanyl, a potent β-arrestin biased agonist, activates the μ-opioid receptor (μOR).* ***The resulting fentanyl-bound pose provides rational insight into a wealth of historical structure-activity-relationship on its chemical scaffold. We found that fentanyl and the synthetic opioid peptide DAMGO require M153 to induce β-arrestin coupling, while M153 was dispensable for G protein coupling. We propose and validate a mechanism where the n-aniline ring of fentanyl mediates μOR β-arrestin through a novel M153 "microswitch" by synthesizing fentanyl-based derivatives that exhibit complete, clinically desirable, G protein biased coupling.*** *Together, these results provide molecular insight into fentanyl mediated β-arrestin biased signaling and a rational framework for further optimization of fentanyl-based analgesics with improved safety profiles.*

As evident from the comparison above, the review from Reviewer #1 is mainly a copy-paste of the abstract, without changing the grammar of the sentence "We propose and validate...". I apologize to the Editors if I seem to be intervening in their work, but I would argue that that review cannot be considered as substantial and informative for the purpose of assessing the quality of this paper. Therefore I would recommend that at least one other review should be obtained before considering publication.

Now about the replies to my initial review. The authors have replied to some of my remarks, but the manuscript was only completed accordingly for a minority of the points. As a reviewer, my task is not to get answers in private, but to make sure that the necessary information to make the work intelligible and reproducible is included in the manuscript.

Below I recall the main points of my initial review and the author responses.

About the biasing coordinates: first it is not clear to me what these coordinates are. The text describes reference points that could define center-of-mass distances, but then the coordinates are described as RMSDs, although I cannot see what are the reference coordinates for such an RMSD.

[authors' reply] We agree with the reviewer and have added the following section to our results to clarify the C.V.s

[The complete section in the manuscript (p.5 l.123) reads:]
The ligand-receptor conformation space was projected onto a 2D collective variable (CV) space comprising two root-mean-squared deviations (RMSDs) where the ligand was decomposed into its flexible isopropylamino (CV1) and rigid carbazole moieties (CV2; Figure S1). The reference points for CV1 and CV2 RMSDs are each a single center-of-geometry, both below the orthosteric site, determined by Cα atoms within TM2/3/7 and TM3/5/6 residues, respectively.

This revised text is still not clear: I am unable based on this paragraph to write down the expression of those collective variables. How can the "reference point" of a RMSD be a single center of geometry?

From the Results:
"Importantly, the underlying mABP is blinded to any exogenous information including the crystallographic ligand position"
Judging from Figure S1a and S1b, that statement is implausible, as each group of the ligand is paired with a suitable part of the binding site, which is clear from the PMFs, as the optimal bound pose coincides with the smaller values of both CVs. The conclusion from that is clear: the correct bound pose is, at least in part, encoded in the choice of CVs. It is unclear how simulations with a more naive choice of CVs would fare.

While the CV is blinded to the correct answer, as in it does not know the correct crystallographic poses, it does make use of the orthosteric site as the known pocket for ligand binding for all orthosteric ligands that modulate GPCR activity.

Those variables (as much as I can judge in the absence of a precise description, see point above) are based on the knowledge of which group of the ligand interacts with which group of the protein, that is, the precise location of the binding site combined with the orientation of the ligand. **That can definitely not be called "blinded to any exogenous information"** (p.5 l.126 of revised text).

To model an alternative CV choice, we reprocessed the carazolol, BU72, and fentanyl mABP simulations and recomputed unweighted histograms where the references for CV1 and CV2 were switched. While this approach is not statistically correct as the histogram is not properly weighed by the mABP, it does provide an approximate answer to the concerns raised above by showing that even if we switch the CV references, the ligand bound-state is at low CV values. This is because the ligand is in the pocket, and nothing more. All atomic details of the pose are resolved by sampling, not the CV choice.

1) The fact that the binding pose is at low CV values in this alternate set of CVs does not say much about whether mABP sampling would work well in this CV space.
2) No matter what happens in other CV spaces, it remains that the CVs that were actually used for this study contained some information about the binding pose, as discussed above. The message in this reply is "it would work with less informative CVs" (which is debatable), but what the manuscript does say is that the original CVs are not informative, which is false.

Given a total simulated time of 76 µs, or about 25 µs per ligand, and prior knowledge of the location of the binding site (as encoded in the choice of collective variables), it is quite likely that

brute force local sampling of the site combined with classic binding free energy estimators would be more cost-effective.

In summary, this paper combines valid and interesting biological results with an attempt to “sell” a numerical method by downplaying its requirements – most notably the choice of collective variables, which are still as of this revision neither precisely documented nor acknowledged as coming from previous knowledge of the binding mode (the “blinded” language noted above). This omission, which persists in the revision despite my initial remarks, is problematic, and cannot be published as is.
