# Supplementary material for: Molecular mechanisms of fentanyl mediated β-arrestin biased signaling
Source: PLoS Comput Biol. 2020 Apr 10;16(4):e1007394. doi: 10.1371/journal.pcbi.1007394 (PMC7176292; doi:10.1371/journal.pcbi.1007394)
Supplement: Attachment — Submitted filename: de_Waal_fentanyl_cover_letter_rev.docx [file pcbi.1007394.s018.docx]

November 22^th^, 2019

**Dear Bert L. de Groot and Nir Ben-Tal,**

Please find attached our revised manuscript for your consideration for publication in *PLOS Computational Biology.* We have modified the manuscript as per the editorial requirements and review suggestions with changes indicated by blue text. Below are our itemized responses to the reviewer comments in **bold** and our responses in blue.

**Reviewer #1: This is very important and well written report describing in the first time proper structure-based drug-design of GPCR biased agonists. Such ligands hold high therapeutic potential as being more efficient and clean from side-effects. It is very promising area however very challenging. The key challenge is to isolate the key features in GPCR binding site responsible for G-protein or β-arrestin signalling/activation pathway (via binding of the agonist). The authors used adaptively biased molecular dynamics simulations to predict which chemical features dictate G protein or β-arrestin signalling. The resulting fentanyl-bound pose provides rational insight into a wealth of historical structure-activity-relationship on its chemical scaffold. Authors found that fentanyl and the synthetic opioid peptide DAMGO require M153 to induce βarrestin coupling, while M153 was dispensable for G protein coupling. We propose and validate a mechanism where the n-aniline ring of fentanyl mediates μOR β-arrestin through a novel M153 “microswitch” by synthesizing fentanyl-based derivatives that exhibit complete, clinically desirable, G protein biased coupling.

Such approach is pioneering and it is great to see that it is working. It is very timely report that would be interesting to wide audience of drug-discovery researches and encourage them to apply such approach in their research.

My recommendation will be to publish it as it is.**

We thank the reviewer for their positive assessment of our work and agree that our manuscript is indeed pioneering, timely, and of substantial interest to a wide audience of drug-discovery researchers.

**Reviewer #2: This manuscript reports a sophisticated and massive simulation study coupled with biochemical bench work on the pharmacology of mu-opioid receptor modulators, all the way to proposing new compounds of pharmacological interest. The scientific results are definitely interesting. However, the simulation methodology is oversold (notably by downplaying its cost and requirements), and the writing is of insufficient quality for publication at this stage.**

We thank the reviewer for their critical analysis of our work and agree with their assessment about their interest. We agree, in the initial version of the manuscript the computational cost of our mABP implementation was not clearly stated. We have added the following statement at the beginning of our discussion:

“In total, we performed 76 us of mABP simulations on our cluster comprising two nodes with 8 Nvidia GTX 1080 TI GPUs. Running at 110-120 ns/day for the GPCR-ligand binding systems, each simulation required approximately 16-17 days per simulation per GPU to complete for an aggregate human time of approximately 50 days.”

and have added the word “targeted” to the following sentence:

“The mABP approach employed here offers a major advantage over virtual screening for targeted compound screening”

Together, these changes highlight that our mABP method, while expensive, is appropriate and tractable for small ligand binding studies.

We have also gone through the manuscript and fixed numerous typos, and cleaned up sentences to improve its clarity. Thank you for noting these below.

**The abstract and author summary give somewhat different accounts of the mABP simulations:
Abstract: "Here we use adaptively biased molecular dynamics simulations to determine how fentanyl (...) activates the μ-opioid receptor"
Author summary: "we use advance [sic] molecular dynamics techniques to obtain the bound geometry of mu-opioid receptor with fentanyl (and derivatives)"
Here the summary is more precise than the abstract, which suggests that the simulations say something about activation, which as far as I know they don't.**

We agree with the reviewer and have removed the word activation from the abstract. Further, we have modified the author summary to reflect these changes.

**About the biasing coordinates: first it is not clear to me what these coordinates are. The text describes reference points that could define center-of-mass distances, but then the coordinates are described as RMSDs, although I cannot see what are the reference coordinates for such an RMSD.**

We agree with the reviewer and have added the following section to our results to clarify the C.V.s

“The reference points for CV1 and CV2 RMSDs are each a single center-of-geometry, both below the orthosteric site, determined by Cα atoms within TM2/3/7 and TM3/5/6 residues, respectively.”

**From the Results:
"Importantly, the underlying mABP is blinded to any exogenous information including the crystallographic ligand position"
Judging from Figure S1a and S1b, that statement is implausible, as each group of the ligand is paired with a suitable part of the binding site, which is clear from the PMFs, as the optimal bound pose coincides with the smaller values of both CVs. The conclusion from that is clear: the correct bound pose is, at least in part, encoded in the choice of CVs. It is unclear how simulations with a more naive choice of CVs would fare.**

While the CV is blinded to the correct answer, as in it does not know the correct crystallographic poses, it does make use of the orthosteric site as the known pocket for ligand binding for all orthosteric ligands that modulate GPCR activity.

To model an alternative CV choice, we reprocessed the carazolol, BU72, and fentanyl mABP simulations and recomputed unweighted histograms where the references for CV1 and CV2 were switched. While this approach is not statistically correct as the histogram is not properly weighed by the mABP, it does provide an approximate answer to the concerns raised above by showing that even if we switch the CV references, the ligand bound-state is at low CV values. This is because the ligand is in the pocket, and nothing more. All atomic details of the pose are resolved by sampling, not the CV choice.

Here, we show the recomputed unweighted histograms for the CVs used in simulation, the switch reference CVs, and a structural overlay for the most probable ligand configuration. In structural snapshots below, the crystal structure, if available, is drawn in white, the CVs used in the manuscript are drawn in blue, and the switched reference CVs are drawn orange.

From this analysis, we see that the carazolol position is shifted slightly compared to the crystal structure and simulated CVs. However, in this pose the ligand forms the expected hydrogen binding network with the conserved aspartic acid. For BU72 and fentanyl, the pose is identical for both CV selections.

**Since the Discussion mention the benefits of this computational approach over virtual screening, it should offer the basis of a fair comparison by stating its total computational cost. The reported mABP simulations cover many tens of microseconds, and the calibration simulations are mentioned but not described in any quantitative detail. What is the total computational cost of the study?**

As stated above, we agree with the reviewer and have included the total simulation time and computational cost in both compute and human time in the manuscript. The total simulation time was approximately 50 human days, of this time, ~16 days were spent on the calibration simulations, ~16 days were spent on the fentanyl and carfentanil docking, and a subsequent ~16 days was spent on lofentanil.

**Accurate modeling of the ligand seems essential for this study, and yet, ligand force field parameters from CGenFF were apparently taken at face value without critical evaluation. CGenFF does provide penalties that give feedback on the expected accuracy of the parameters. It would be interesting to read about those. It would seem wasteful to use a sophisticated sampling method to sample a force field of unknown accuracy. A point of detail here: the team beahind CGenFF made the highly unfortunate and confusing choice of calling the parameter assignment program the same name as the force field itself. To decrease the confusion slightly, this manuscript could say the parameters were assigned by the CGenFF *program*.**

We agree with the reviewer that accurate modelling of ligands is an import step of any computational study. However, we disagree that the accuracy of using the CGenFF webserver for ligand parameterization was of detriment to our study as we provided empirical tests of our in silico derived hypotheses. Here, we provide the penalties for each ligand simulated in our study:

RESI CARA	1.000 ! param penalty= 89.000 ; charge penalty=	9.734

RESI BU72	1.000 ! param penalty= 401.100 ; charge penalty= 84.521

RESI FEN	1.000 ! param penalty= 91.500 ; charge penalty= 43.272

RESI CAR	1.000 ! param penalty= 91.500 ; charge penalty= 45.100

RESI LOF	1.000 ! param penalty= 91.500 ; charge penalty= 43.272

From these penalties, we see that CGenFF was able to accurately describe complex ligands, such as BU72 that comprise a fused heterocyclic scaffold. More importantly, the validity of our simulations was tested experimentally by producing and testing novel compounds.

We agree with the reviewer about confusion over CGenFF and have modified the text to include the CGenFF program to avoid confusion.

**Fig. 1a's caption is insufficient. One might guess that "30k" etc. indicate the number of atoms in each resepctive benchmark, but that should be spelled out in the figure or caption. The benchmarks should also be documented in the methods section, where there is no mention whatsoever of them.**

We apologize for omitting this information from the figure legend and have modified it accordingly. Also, we have added the appropriate section in the methods describing the benchmark system.

**The Methods state that non-bonded interactions were cut at 8 Angstrom. I assume an approximate long-range LJ term was introduced, this should be stated. Depending on exactly what type of long-range expression is used, it may not be accurate for strongly anisotropic systems like this.**

Within AMBER, the default simulation settings include a continuum term for interactions beyond the cutoff. We agree with the reviewer’s assessment this may not be accurate for anisotropic systems like ours, we disagree that it had any negative impact on our results as evident of correct identification of ligand bound poses and generation of a actionable hypothesis that was subsequently investigated and validated through biochemical and ligand design/synthesis experiments. All the inputs for ligand binding simulation can be found at the GitHub repo associated with this work. ( <https://github.com/ParkerdeWaal/fABAMBER> )

**Specific comments
As the manuscript features no page (let alone line) numbers, my references to the text will be approximate.
I started collecting typos and then gave up. Please run this through some form of spell check, and a good proofreading for grammar and syntax.**

We have added pages and line numbers to the manuscript. In addition, we have gone through and proofread the manuscript for grammar and syntax.

**Author list:
No author is indicated as affiliated to institution #3.**

Thank you for identifying this omission! We have fixed it accordingly.

**Results:
*"GROAMCS"**

Fixed.

*"rotomer", *"rotomeric" (many instances)

Fixed.

**Methods:
*"atomospheric" I suppose that means atmospheric, but then it's a strange turn of phrase. Please clarify.**

We have corrected the methods to read atmospheric.

**What do the "5000 rounds of refinement" with Modeller refer to specifically?**

5000 founds of refinement refers to the number of models generated. We have replaced the word “refinement” with “model generation” to clarify this process.

**The text should explain the acronym ICL3 for the benefit of non-GPCR-specialist readers.**

Agreed, we now explicitly state what the ICL3 is.

**The sentence "Substantial pharmacophore overlap..." is missing a verb.**

We have added the missing verb.

As part of the editorial requirements, the following change was made to the manuscript:

**-We noticed that you used “data not shown” in the manuscript, we do not allow these references. PLOS Computational Biology data access policy requires that all data be either published with the manuscript or made available in a publicly accessible database. Please amend the supplementary material to include the referenced data or remove the references. Please note these changes in the Response to reviewers**

We have removed the references to “data not shown” in the manuscript with regards to the overfill calibration simulations. Trajectory “spoiling” resulting from visiting high-energy regions resulting in irreversible changes is well documented in the enhanced sampling literature, thus motivating our use of overfill protection. As per the data availability statement, all trajectories – including the overfill calibration simulations, will be hosted on our internal webserver and will be made readily available upon request.

We thank the reviewers for their diligent review of our manuscript

Sincerely,

|  |  |
| --- | --- |
| Bradley M. Dickson, Ph.D. | H. Eric Xu, Ph.D. |
