# Supplementary material for: Molecular mechanisms of fentanyl mediated β-arrestin biased signaling
Source: PLoS Comput Biol. 2020 Apr 10;16(4):e1007394. doi: 10.1371/journal.pcbi.1007394 (PMC7176292; doi:10.1371/journal.pcbi.1007394)
Supplement: Attachment — Submitted filename: de_waal_cover_letter_rev_2.docx [file pcbi.1007394.s020.docx]

February 12^th^, 2020

**Dear Bert L. de Groot and Nir Ben-Tal,**

Please find attached our revised manuscript for your consideration for publication in *PLOS Computational Biology.* We have modified the manuscript as per the editorial requirements and review suggestions with changes indicated by blue text. Below are our itemized responses to the reviewer comments in **bold** and our responses in blue.

Upon review of our manuscript, we found an error in our chemical drawing of FD-1. Originally, the R- group was an “ethyl”, but should have been a “methyl” group. This has been changed in Figure 6 and has no impact on our findings as the rank-order for ligands by size remains the same. We apologize for this oversight.

**As a preliminary note, I must compare the review sent by Reviewer 1 and the abstract of the manuscript:**

**…**

**As evident from the comparison above, the review from Reviewer #1 is mainly a copy-paste of the abstract, without changing the grammar of the sentence “We propose and validate...”. I apologize to the Editors if I seem to be intervening in their work, but I would argue that that review cannot be considered as substantial and informative for the purpose of assessing the quality of this paper. Therefore I would recommend that at least one other review should be obtained before considering publication.**

We refer to the Editors discretion on this matter as we do not know the expertise or the subject matter field of reviewer #1.

**Now about the replies to my initial review. The authors have replied to some of my remarks, but the manuscript was only completed accordingly for a minority of the points. As a reviewer, my task is not to get answers in private, but to make sure that the necessary information to make the work intelligible and reproducible is included in the manuscript.**

PLOS Computational Biology allows publication of referee-author correspondence. Thus, our exchanges are not private. Toward reproducibility, the source code for our simulations and simulation inputs have been publicly available on GitHub since this manuscript was submitted. We refer to the manuscript for the direct link.

**Below I recall the main points of my initial review and the author responses.**

**…**

**This revised text is still not clear: I am unable based on this paragraph to write down the expression of those collective variables. How can the "reference point" of a RMSD be a single center of geometry?**

We will write the CV below for clarity but we note that the CV has been explicitly documented and publicly available since the manuscript was submitted. The source code is available on GitHub and is documented with comments. For example, the CV is defined in the source code here:

<https://github.com/ParkerdeWaal/fABAMBER/blob/master/AMBER16/fentanyl.diff#L516>

The RMSD can be written, referenced to a single center of geometry (COG), as

where we use $i$ to index atoms and $j$ to index dimension. $q_{i,j}$ is an atomic coordinate and ${COG}_{j}$ is the COG. All $n$particles are referenced to a single COG. We do not write this explicitly in the manuscript because it is explicit in the source code.

**Those variables (as much as I can judge in the absence of a precise description, see point above) are based on the knowledge of which group of the ligand interacts with which group of the protein, that is, the precise location of the binding site combined with the orientation of the ligand. That can definitely not be called “blinded to any exogenous information” (p.5 l.126 of revised text).**

As noted above, the CVs have been available in the source code since our initial submission and described with a graphical representation in our supplemental figures. We agree with the reviewer and have removed our language referring to our CVs as blinded for carazolol and BU72 as they could be seen as tailored to match the orientation of their crystallographic poses. As such, we have updated our language in the manuscript to remove the following text at p.5 l.123 & 126:

Importantly, the underlying mABP is blinded to any exogenous information including the crystallographic ligand position and simulations were not manually stopped once the correct pose was found. … Simulations do make use of the fact that ligands bind within the orthosteric site.

And have added the following to the end of our first results section at p.5 l.149:

As the CVs used above can be viewed as tailored to carazolol and BU72, we also carried out a series of simulations where the COG reference points were switched to explore how CV construction effects sampling efficiency (see Figure S1b and imagine switching colors of the reference points). Across 4 independent simulations for each ligand, no binding events were observed in the carazolol simulations whereas one BU72 simulation identified a local minima within the orthosteric site (Figure S11). This simulation, however, failed to correctly identify the crystallographic conformation of BU72 suggesting that this alternative CV choice is less efficient than the original construction. Further comment on this pairing is made below.

Our original manuscript said “blinded” because we guessed which way to pair the two COG with different moieties of fentanyl. We have added text to the manuscript to explain how this guess would be correct nearly 50% of the time, due to symmetry in the collective variable. This discussion can be found at p.10 l.336:

With any method requiring construction of collective variables, one accepts that sampling efficiency is dependent on making a good construction. In the present case, the collective variable has a symmetry that can be realized by exchanging identities (“blue” and “red” in Figure S1b) of the two RMSD reference points. Our particular construction appears to be the best one, evidenced by the significant agreement among our many repeated simulations and the fact that the blue CV atoms in Figure S3b ended up nearest the blue reference point. The 50/50 odds played out in our favor here, but in general one could explore both CV constructions with trial simulations.

A poorly constructed CV decreases the efficiency of the mABP method but the proportionality of this decrease is completely unknown and entirely dependent on the application. We report in SI that switching the references in our simulations does reduce efficiency (Figure S11). Unfortunately it is impossible to quantify the efficiency loss. Through comparison to data generated by large-scale brute force simulation using Anton [25], we estimate our mABP approach with good CV to be anywhere from 8 to 18 times more efficient when comparing simulations of ligand binding to β2AR (Supplemental Table II). Without running the alternate CV for at least 8 times longer than our production trajectories, we cannot assess the efficiency. The reversed CV are less efficient, but it is infeasible to measure this efficiency.

Lastly, we have added text to explain our CV selection for the fentanyl series at p6. l.163 that reads:

Atoms comprising the n-alkyl phenyl ring were assigned to CV1 whereas atoms within piperidine scaffold, propyl-amide, and n-aniline ring were assigned to CV2 without any further experimentation or justification

**1) The fact that the binding pose is at low CV values in this alternate set of CVs does not say much about whether mABP sampling would work well in this CV space.**

**2) No matter what happens in other CV spaces, it remains that the CVs that were actually used for this study contained some information about the binding pose, as discussed above. The message in this reply is “it would work with less informative CVs” (which is debatable), but what the manuscript does say is that the original CVs are not informative, which is false.**

Agreed. We ran simulations with the reference points of the CV switched to try and estimate the impacts on sampling efficiency as noted above. Unfortunately it is not possible to run long enough to determine the efficiency exactly. All we can report is that the alternate CV are less efficient than our original choice. We estimate, based on simulations that used the Anton computer, that our original CV choice allowed mABP to operate anywhere from 8 to 18 times more efficiently than brute force simulation (comparing our carazolol to available B_2_AR simulations). We cannot run 8 times longer than out original production runs to measure the efficiency, but we tried running for the same length as our production runs. We report the data in SI, the simulations do not correctly pose carazolol or BU72. Thus, we know the efficiency is worse but we do not know how much worse.

We have attempted to measure efficiency, addressing referee-2 point (1). We have adjusted the text to reflect exactly the risk we took in building the CV and made it clear that we did not know how the fentanyl series would bind. This change, and our attempt to measure efficiency for the alternate CV addresses point (2).

**Given a total simulated time of 76 μs, or about 25 μs per ligand, and prior knowledge of the location of the binding site (as encoded in the choice of collective variables), it is quite likely that brute force local sampling of the site combined with classic binding free energy estimators would be more cost-effective.**

As reported in the manuscript and detailed within the supplemental table describing our simulations, we ran carazolol for a total of 32 μs, BU72 for a total of 8 μs, and the fentanyl analogs for a total of 12 μs each. This averages to 15.2 μs per-ligand. Including the recently simulated switched CV simulations for carazolol and BU72, raising their totals to 40 μs and 16 μs respectively, the average simulation time per-ligand is now 18.4 μs. This number is still well below the “25 μs per ligand” noted by the referee above.

To the referee’s point about relative efficiency between brute force local sampling, We compared to data generated by Anton for B2AR ligand binding simulations and found an 8 to 18 fold efficiency advantage for mABP. The efficiency of ligand binding events is reported in supplemental table II. Whether or not some other method could also achieve this efficiency is unknown to us and we’ve made no statements in the manuscript regarding such a speculation.

Further, we note that recent unbiased simulations of TRV-130 binding to the mouse mu opioid receptor, totaling 44.25 μs, failed to identify a final pose where the ligand’s protonated amine formed a salt bridge with D147. (see <https://pubs.acs.org/doi/full/10.1021/acs.biochem.6b00948> ). As we note in our manuscript, this is a conserved, critical interaction that is required to both anchor the ligand and activate the receptor that is seen in all crystal structures published to date We have added this point to our discussion about the relative efficiency vs unbiased simulations at p.10 l.325:

We note that recent attempts to “dock” fentanyl to the μOR crystal structure and long-timescale simulations of TRV-130, a recently described μOR agonist totaling over 44 μs of simulation, did not observed this key interaction ~~conserved for class A biological amine receptors~~ [39, 40].

**In summary, this paper combines valid and interesting biological results with an attempt to “sell” a numerical method by downplaying its requirements – most notably the choice of collective variables, which are still as of this revision neither precisely documented nor acknowledged as coming from previous knowledge of the binding mode (the “blinded” language noted above). This omission, which persists in the revision despite my initial remarks, is problematic, and cannot be published as is.**

We thank the reviewer for their comments regarding our valid and interesting biological results. In summary, we show that (1) the CV have always been well documented as they were explicitly reported in the publicly available source code and (2) cleared up the fact that, while the pose of some of the ligands is known, the pose of the fentanyl series was unknown. We made use of the binding pocket and took a 50/50 risk on assigning references for the CV. We guessed correctly, and have added what we could to report what happens if the incorrect choice is made. We also suggested exploration of CV symmetries to mitigate this risk.

We thank the reviewers for their review of our manuscript.

Sincerely,

|  |  |
| --- | --- |
| Bradley M. Dickson, Ph.D. | H. Eric Xu, Ph.D. |
